# Hierarchical Heterostructures of NiCo_2_O_4_@XMoO_4_ (X = Ni, Co) as an Electrode Material for High-Performance Supercapacitors

**DOI:** 10.1186/s11671-016-1475-9

**Published:** 2016-05-18

**Authors:** Jiyu Hu, Feng Qian, Guosheng Song, Linlin Wang

**Affiliations:** No. 2 High School of East China Normal University, Shanghai, 201203 China; Institute of Functional Nano & Soft Materials (FUNSOM), Collaborative Innovation Center of Suzhou Nano Science and Technology, Soochow University, Suzhou, Jiangsu 215123 China; College of Chemistry and Chemical Engineering, Shanghai University of Engineering Science, Shanghai, 201620 China

**Keywords:** Supercapacitor, NiCo_2_O_4_@XMoO_4_, Heterostructures, Nanosheet arrays

## Abstract

Hierarchical heterostructures of NiCo_2_O_4_@XMoO_4_ (X = Ni, Co) were developed as an electrode material for supercapacitor with improved pseudocapacitive performance. Within these hierarchical heterostructures, the mesoporous NiCo_2_O_4_ nanosheet arrays directly grown on the Ni foam can not only act as an excellent pseudocapacitive material but also serve as a hierarchical scaffold for growing NiMoO_4_ or CoMoO_4_ electroactive materials (nanosheets). The electrode made of NiCo_2_O_4_@NiMoO_4_ presented a highest areal capacitance of 3.74 F/cm^2^ at 2 mA/cm^2^, which was much higher than the electrodes made of NiCo_2_O_4_@CoMoO_4_ (2.452 F/cm^2^) and NiCo_2_O_4_ (0.456 F/cm^2^), respectively. Meanwhile, the NiCo_2_O_4_@NiMoO_4_ electrode exhibited good rate capability. It suggested the potential of the hierarchical heterostructures of NiCo_2_O_4_@CoMoO_4_ as an electrode material in supercapacitors.

## Background

To meet the increasing requirement for portable electronics, hybrid electronic vehicles and other micro- and nanodevices, numerous studies have been carried out to develop many kinds of energy storage systems. As an important energy storage device, the widely studied supercapacitors, also known as electrochemical capacitors, have been believed as a promising candidate due to their high specific power, long cycling life, fast charge and discharge rates, and reliable safety [1–9]. Though these supercapacitors demonstrated these distinctive advantages, as compared with the batteries and fuel cells, the relatively lower energy densities seriously block their large-scale practical application [4, 10]. So far, various electrode materials which include carbon materials [11, 12], transition metal oxides [2, 13–15], and conducting polymers [16, 17] have been designed and synthesized to enhance the electrochemical properties for the practical applications in the supercapacitors.

Recently, some bimetallic oxides, such as NiCo_2_O_4_ [15, 17–20], ZnCo_2_O_4_ [21, 22], NiMoO_4_ [23], and CoMoO_4_ [24, 25], have been developed as a new electrode material used for supercapacitors because of their excellent electrical conductivity and multiple oxidation states (as compared with the binary metal oxides) for reversible Faradaic reactions [26]. For fully utilizing the advantages of active materials and thus optimizing the performance of these materials, plenty of efforts has been devoted, i.e., realizing additive/binder-free electrode architectures, which eliminate the “dead surface” and release complicated process in traditional slurry-coating electrode and meaningfully improve the utilization rate of electrode materials even at high rates [4, 27], constructing 3D hierarchical heterostructures, which can provide efficient and fast pathways for electron and ion transport [20, 28], and exploring smart integrated array architectures with rational multi-component combination, which can achieve the synergistic effect from all individual constituents [29–31]. Taken some successful examples, Co_x_Ni_1 − x_DHs/NiCo_2_O_4_/CFP composite electrodes were prepared by a hydrothermal route and an electrodeposition process, showing high capacitance of ∼1.64 F/cm^2^ at 2 mA/cm^2^, good rate capability, and excellent cycling stability [20]; 3D hierarchical NiCo_2_O_4_@NiMoO_4_ core-shell nanowire/nanosheet arrays delivered a high areal capacitance of 5.80 F/cm^2^ at 10 mA/cm^2^, excellent rate capability, and high cycling stability [32]. Despite these notable achievements, it is still a hard task to design and construct 3D hierarchical heterostructures made of the bimetallic oxides with improved electrochemical properties for the supercapacitors.

Herein, we report hydrothermal growth of hierarchical heterostructures of NiCo_2_O_4_@XMoO_4_ (X = Ni, Co) as an electrode material for the supercapacitors with improved performances. Within these hierarchical heterostructures, high electrochemical activity of NiCo_2_O_4_ not only shows outstanding pseudocapacity but also can be regarded as a backbone to provide reliable electrical connection to the XMoO_4_ (X = Ni, Co). Between them, the NiCo_2_O_4_@NiMoO_4_ electrode material showed a highest areal capacitance of 3.74 F/cm^2^ at 2 mA/cm^2^, which was much higher than the NiCo_2_O_4_@CoMoO_4_ electrode material (2.452 F/cm^2^), and good rate capability, implying its prospect as an alternative electrode material in the supercapacitors.

## Methods

### Synthesis of NiCo_2_O_4_@XMoO_4_ (X = Ni, Co) Composite Nanosheet Arrays

All the reactants here were analytically graded and used without further purification. The synthesis of the composite nanosheet arrays was described briefly as follows: Firstly, the NiCo_2_O_4_ nanosheet arrays were grown on the Ni foam according to a reference [17]. Secondly, the product of as-grown NiCo_2_O_4_ nanosheet arrays was put into a 60-mL Teflon-lined autoclave, which contained 0.5 mmol of NiCl_2_·6H_2_O (or CoCl_2_·6H_2_O), 0.5 mmol of Na_2_MoO_4_·2H_2_O, and 50 mL of deionized water. The autoclave was sealed and maintained at 120 °C for 2 h (or 1 h) in an electric oven and then cooled down to room temperature. The NiCo_2_O_4_@XMoO_4_ (X = Ni, Co) composites on the Ni foam were carefully washed with deionized water and absolute ethanol, successively, and then dried at 60 °C overnight. Lastly, the samples were annealed at 400 °C for 1 h at a ramping rate of 1 °C/min.

### Material Characterizations

As-synthesized products were characterized by means of a D/max-2550 PC X-ray diffractometer (XRD; Rigaku, Cu-Kα radiation), a scanning electron microscopy (SEM; S-4800), and a transmission electron microscopy (TEM; JEM-2100 F) equipped with an energy-dispersive X-ray spectrometer (EDX).

## Results and Discussion

**Fig. 1:**
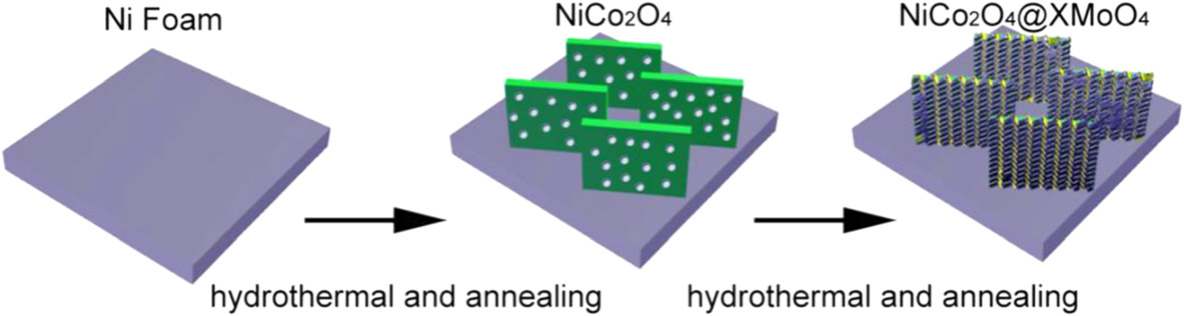
Schematic depicting the growth process of the hierarchical heterostructures of the NiCo_2_O_4_@XMoO_4_ (X = Ni, Co)

In this work, the NiCo_2_O_4_@XMoO_4_ (X = Ni, Co) hierarchical heterostructures were successfully synthesized for electrode materials. As depicted schematically in Fig. 1, the synthesis process includes two steps: the hydrothermal growth of NiCo_2_O_4_ nanosheets on the Ni foam and subsequent annealing as the first step and the hydrothermal growth of NiMoO_4_ or CoMoO_4_ nanosheets (coatings) on the NiCo_2_O_4_ nanosheet arrays and another annealing process as the second step. Herein, 3D Ni foam, with uniform macropore structure, huge supporting area, and high electrical conductivity, was selected as a current collector for the growth of electrode materials, which can provide efficient electrolyte penetration to enable fast ion diffusion [27, 33]. Meanwhile, the NiCo_2_O_4_ nanosheets grown uniformly on Ni foam functioned as the backbone to support and give reliable electrical connection to XMoO_4_ (X = Ni, Co) nanosheets, which can contribute to electronic and ionic diffusion and improve the utilization rate of electrode material. More importantly, the NiCo_2_O_4_ electrode material with high electrochemical activity can also act as active materials for charge storage and contribute to the capacitance.

**Fig. 2:**
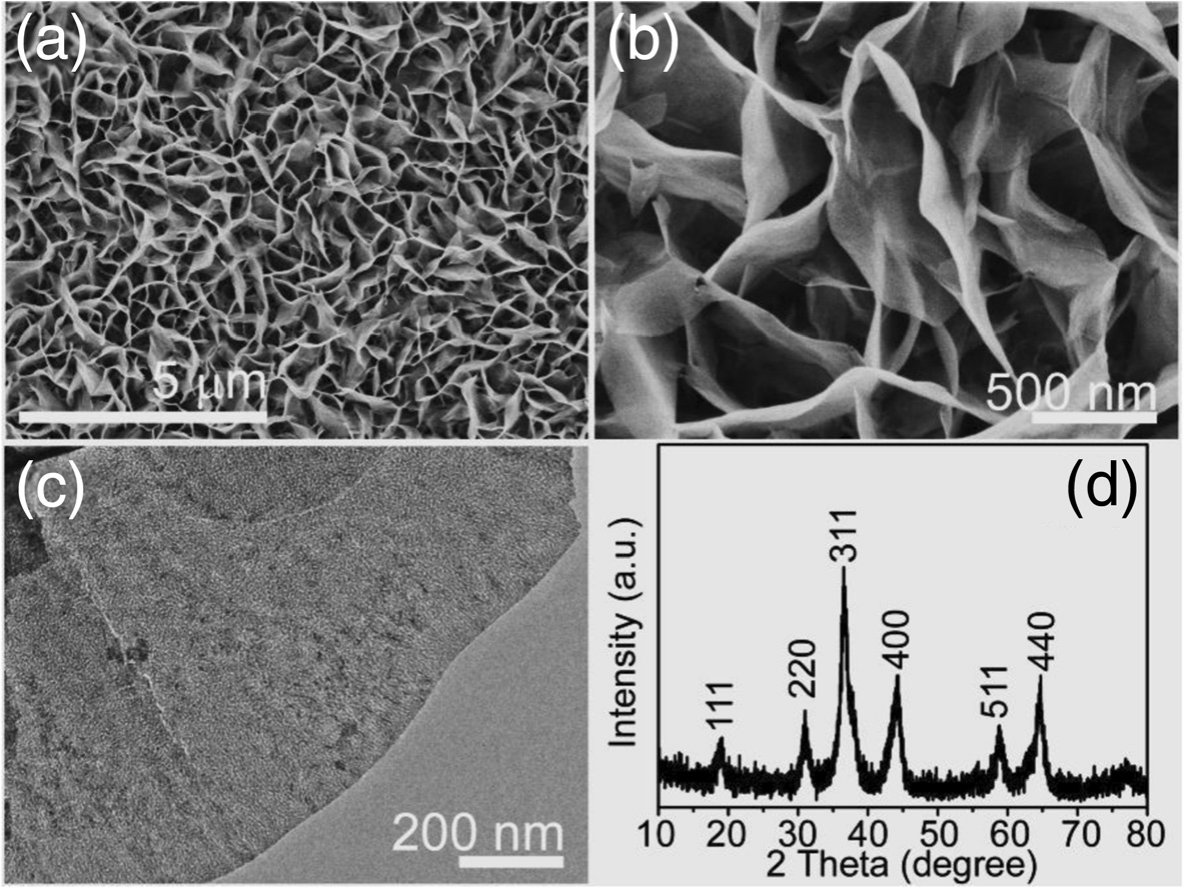
**a**, **b** Low- and high-magnification SEM images of as-prepared NiCo_2_O_4_ nanosheet arrays on the Ni foam. **c** TEM image of a NiCo_2_O_4_ nanosheet. **d** XRD pattern of the product scratched from the Ni foam

Combining the hydrothermal reaction and the annealing process resulted in the NiCo_2_O_4_ nanosheet arrays grown on Ni foam. Detailed morphology and microstructure of the NiCo_2_O_4_ nanosheets were investigated via the scanning electron microscopy (SEM) and transmission electron microscopy (TEM). Figure 2a, b shows the highly tight NiCo_2_O_4_ nanosheets (with a thickness of ~30–50 nm) that grew uniformly and vertically on the Ni foam and interconnected with each other, resulting in a highly porous structure with an abundant open space. Figure 2c shows a NiCo_2_O_4_ nanosheet almost transparent to electron beam, suggesting an ultrathin feature. Intriguingly, numerous mesoporous arrays are distributed uniformly throughout the whole NiCo_2_O_4_ nanosheet. The nanosheet arrays were scratched from the Ni foam and were then characterized by X-ray diffraction (XRD) to determine the crystalline phase of the product. As shown in Fig. 2d, all well-defined diffraction peaks can be indexed to the cubic phase NiCo_2_O_4_ by referring to the JCPDS card (no. 20-0781).

**Fig. 3:**
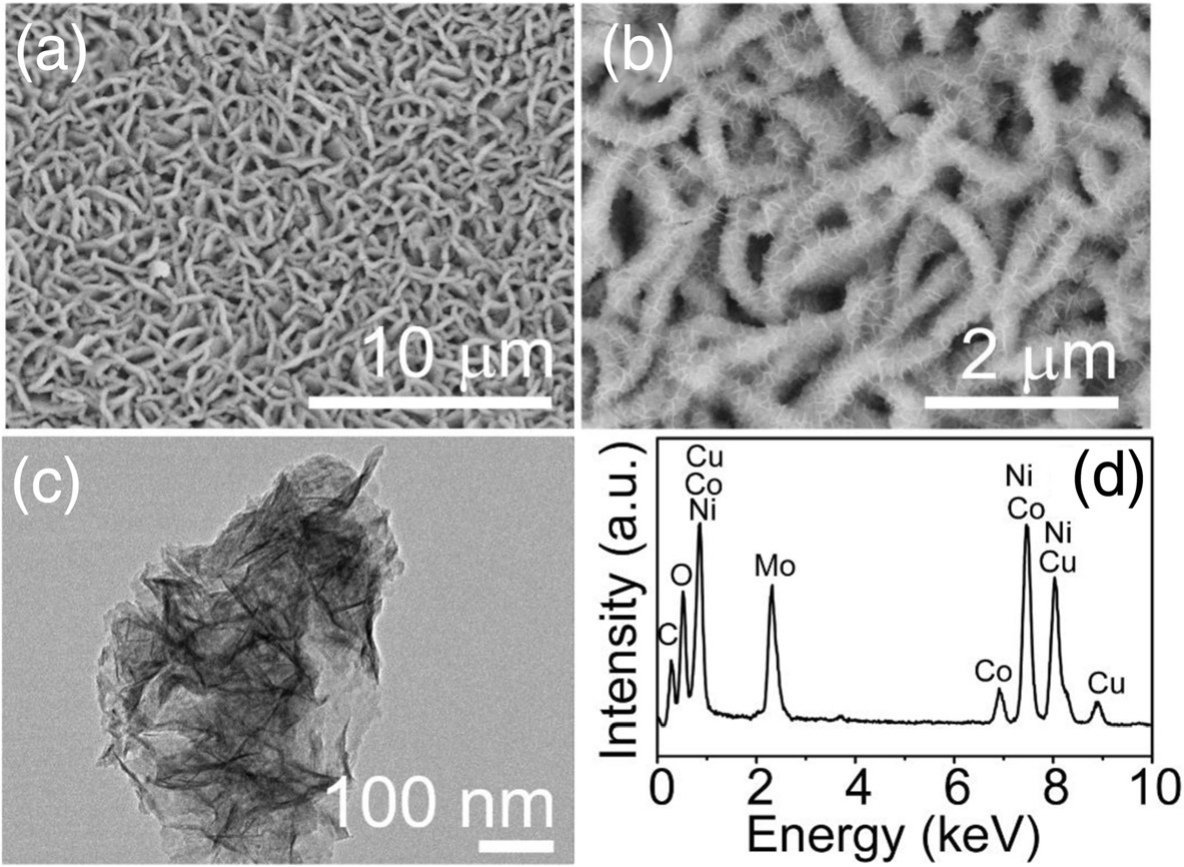
**a**, **b** Low- and high-magnification SEM images of the hierarchical heterostructures of the NiCo_2_O_4_@NiMoO_4_ composite nanosheet arrays on the Ni foam. **c** TEM image and (**d**) EDX spectrum of the NiCo_2_O_4_@NiMoO_4_ composite nanosheet arrays

The NiCo_2_O_4_ nanosheet arrays grown on the Ni foam act as an ideal scaffold to load additional electroactive pseudocapacitive materials, thus enhancing the electrochemical performance. Considering this merit, NiMoO_4_ or CoMoO_4_ nanosheets were grown on the surface of the NiCo_2_O_4_ nanosheets via a hydrothermal reaction and an annealing step similar to the first growth step described above, forming NiCo_2_O_4_@NiMoO_4_ composite nanosheet arrays (a core-shell structure or shaped like caterpillar). Figure 3a, b shows SEM images of the NiCo_2_O_4_@NiMoO_4_ composite nanosheet arrays, in which the ultrathin NiMoO_4_ nanosheets were uniformly grown on the surface of NiCo_2_O_4_ nanosheets and thus plenty of the space among NiCo_2_O_4_ nanosheets is utilized abundantly, and a thickness of the NiCo_2_O_4_@NiMoO_4_ composite nanosheets is in the range of ~250–300 nm. Importantly, the integration of the NiMoO_4_ material into the original NiCo_2_O_4_ nanosheet arrays does not destroy the ordered structure. In addition, these NiMoO_4_ nanosheets are interconnected with each other to form a highly porous morphology, which can provide more active sites for electrolyte ions to transport efficiently. Figure 3c shows the TEM image of the NiCo_2_O_4_@NiMoO_4_ composites. The result shows that the NiMoO_4_ nanosheets are highly dense but still do not cover the entire mesoporous NiCo_2_O_4_ nanosheets fully. Moreover, energy-dispersive X-ray (EDX) spectrum (Fig. 3d) indicates that Ni, Co, Mo, and O can be detected in the composites. Surely, the Cu and C signals come from the carbon-supported Cu grid.

**Fig. 4:**
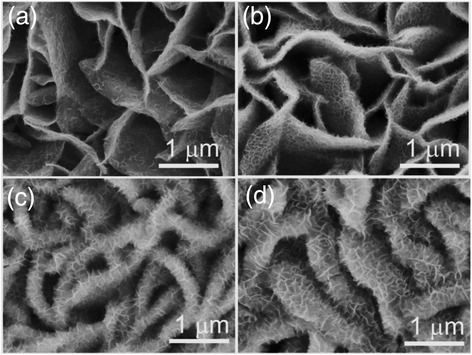
SEM images of the morphology evolution of the NiCo_2_O_4_@NiMoO_4_ nanosheet arrays formed via different reaction time: (**a**) 0.5 h, (**b**) 1 h, (**c**) 2 h, and (**d**) 4 h

Figure 4 shows the SEM images of the samples, prepared via a different hydrothermal reaction time. It is used to demonstrate the formation process of the samples. Figure 4a depicts the NiCo_2_O_4_@NiMoO_4_ nanosheet arrays formed via 0.5 h of the hydrothermal reaction. It can be seen that the nanosheets’ surface of NiCo_2_O_4_ loses their original smooth appearance and was interspersed by many fine NiMoO_4_ nanosheets. Then, the reaction time was extended to 1 h and the SEM image (Fig. 4b) showed the results. Almost all the naked surface observed before was fully coated by NiMoO_4_ nanosheets with the thickness increased to ~100–150 nm. As the time of the hydrothermal reaction becomes longer, the composition becomes thicker and at last the whole thickness in Fig. 4d is ~400–500 nm, which leads to a much smaller interspace among the adjacent sheets. But the growth of mass NiMoO_4_ nanosheets on the NiCo_2_O_4_ nanosheets may decay the utilization of the NiCo_2_O_4_ (core) materials and even some NiMoO_4_ (shell) materials may be blocked from the access to electrolyte. Therefore, the hydrothermal reaction time should be optimized (e.g., 2 h) to get an improved electrochemical properties.

**Fig. 5:**
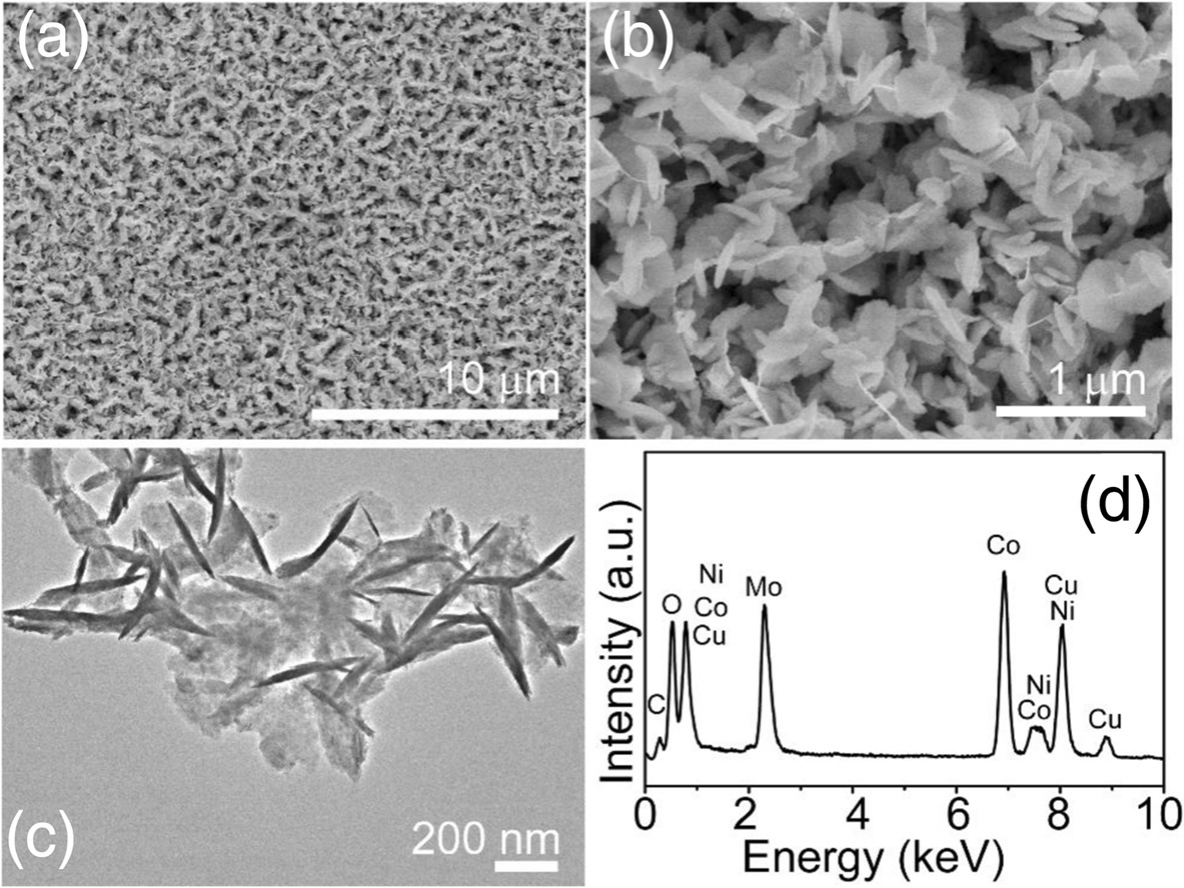
**a**, **b** Low- and high-magnification SEM images and **c** TEM image of the hierarchical heterostructures of the NiCo_2_O_4_@CoMoO_4_ composite nanosheets. **d** EDX spectrum of the NiCo_2_O_4_@CoMoO_4_ composite nanosheet arrays

Hierarchical heterostructures of the NiCo_2_O_4_@CoMoO_4_ composite nanosheets were also fabricated as a comparison with the NiCo_2_O_4_@NiMoO_4_ nanosheet arrays for their usage as an electrode material. Figure 5a, b shows the SEM image of the NiCo_2_O_4_@CoMoO_4_ composite nanosheets. It clearly confirms that the whole surface of the NiCo_2_O_4_ nanosheets is homogeneously covered by the CoMoO_4_ nanosheets, and the uniformity of these structures is similar to that of the NiCo_2_O_4_@NiMoO_4_ nanosheet arrays. As the TEM image (Fig. 5c) demonstrates, the thickness of the CoMoO_4_ nanosheets is about 20–50 nm. Additionally, the composition of the as-synthesized NiCo_2_O_4_@CoMoO_4_ composites was confirmed by EDX. As shown in Fig. 5d, the peaks of Cu and C derive from the Cu grid, and the strong signals of Ni, Co, Mo, and O further ascertain the formation of NiCo_2_O_4_@CoMoO_4_.

**Fig. 6:**
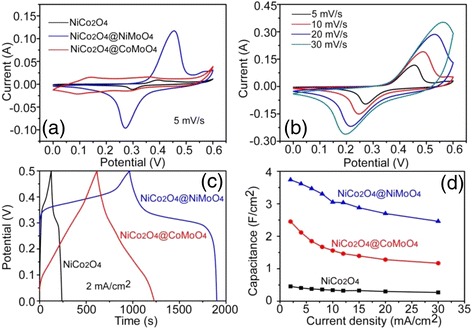
**a** CV curves of the NiCo_2_O_4_@XMoO_4_ (X = Ni, Co) and NiCo_2_O_4_ electrodes at a scan rate of 5 mV/s. **b** CV curves of the NiCo_2_O_4_@NiMoO_4_ electrode at a various scan rate, i.e., 5, 10, 20, and 30 mV/s. **c** CD curves of the NiCo_2_O_4_@XMoO_4_ (X = Ni, Co) and NiCo_2_O_4_ electrodes collected at a current density of 2 mA/cm^2^. **d** Areal capacitance of the NiCo_2_O_4_@XMoO_4_ (X = Ni, Co) and the NiCo_2_O_4_ electrodes calculated from the CD curves as a function of current density

Then, the electrochemical properties of the hierarchical heterostructures of the NiCo_2_O_4_@XMoO_4_ (X = Ni, Co) were investigated to evaluate their applicability as an active material for the supercapacitors, where a three-electrode cell with a saturated calomel electrode (SCE) reference electrode, a Pt counter electrode, and a KOH aqueous electrolyte (3 M) inside was used. As a comparison, the cyclic voltammogram (CV) curves from three electrodes made from NiCo_2_O_4_ and NiCo_2_O_4_@XMoO_4_ (X = Ni, Co) materials, respectively, were shown in Fig. 6a. It was recorded with a potential window ranging from 0 to 0.6 V and a scan rate of 5 mV/s. Deduced from the CV curves’ shape, the Faradaic redox reactions associated with M-O/M-O-OH (M = Ni, Co) dominated the capacitance characteristics [18, 20] of these electrodes. Obviously, the surface area of the NiCo_2_O_4_@NiMoO_4_ electrode is higher than that of the NiCo_2_O_4_@CoMoO_4_ electrode and NiCo_2_O_4_ electrode, suggesting that the NiCo_2_O_4_@NiMoO_4_ electrode possessed a greater capacitance than the other two. The high capacitance of the NiCo_2_O_4_@NiMoO_4_ electrode is mainly attributed to the fact that a highly porous nanostructure that originated from numerous ultrathin NiMoO_4_ nanosheets grown on the NiCo_2_O_4_ nanosheet surface should provide more active sites for increasing electrolyte ion transportation efficiency to enhance the utilization of the whole electrode. Also, the CV curves of the NiCo_2_O_4_@NiMoO_4_ electrodes taken via a various scan rate, i.e., 5, 10, 20, and 30 mV/s, were collected, as shown in Fig. 6b. It was noted that the peak position shifted slightly with the increase of scan rate, implying a good capacitive behavior and a high-rate capability of the electrode material. The galvanostatic charge-discharge (CD) method was applied to compare the capacitive ability of the NiCo_2_O_4_ electrode and two composite electrodes of NiCo_2_O_4_@NiMoO_4_ and NiCo_2_O_4_@CoMoO_4_ at the same current density of 2 mA/cm^2^, as illustrated in Fig. 6c. It was found that the NiCo_2_O_4_@NiMoO_4_ electrode possessed a longer discharging time than the NiCo_2_O_4_@CoMoO_4_ and pure NiCo_2_O_4_ electrodes, demonstrating that such an electrode has an enhanced capacitance. Moreover, the areal capacitance of the electrode materials could be calculated from their CD curves by this equation: *C* = (*I* · *t*)/(*S* · Δ*V*), where *I* (A) is the current for the charge-discharge measurement, *t* (s) is the discharge time, *S* is the geometrical area of the electrode [31], and Δ*V* (V) is the voltage interval of the discharge. As shown in Fig. 6d, the NiCo_2_O_4_@NiMoO_4_ electrode always exhibited higher areal capacitances than the NiCo_2_O_4_@CoMoO_4_ and NiCo_2_O_4_ electrodes. The maximal areal capacitance of the NiCo_2_O_4_@NiMoO_4_ electrode was found to be 3.74 F/cm^2^ at 2 mA/cm^2^, which is much higher than the NiCo_2_O_4_@CoMoO_4_ electrode (2.45 F/cm^2^), and 8 times higher than the NiCo_2_O_4_ electrode (0.46 F/cm^2^). In particular, the NiCo_2_O_4_@NiMoO_4_ electrode still has an areal capacitance of 2.46 F/cm^2^ even if the current density increased to 30 mA/cm^2^, retaining appropriately 66 % of its initial value. However, the NiCo_2_O_4_@CoMoO_4_ electrode and the NiCo_2_O_4_ electrode only showed the areal capacitance of 1.17 and 0.27 F/cm^2^ at a high current density of 30 mA/cm^2^, respectively.

**Fig. 7:**
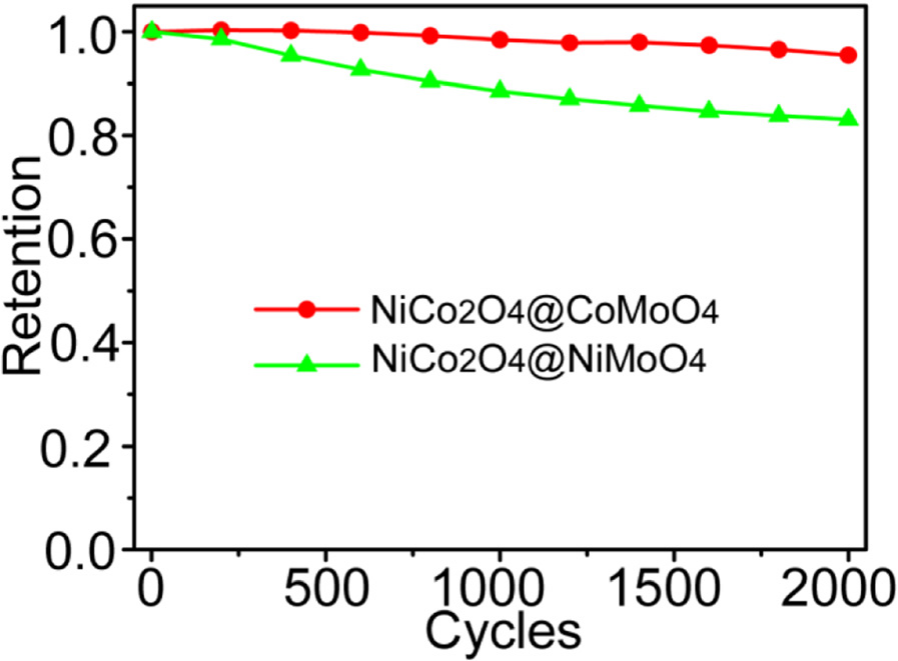
Cycling performance of the NiCo_2_O_4_@XMoO_4_ (X = Ni, Co) composite electrodes at a scan rate of 60 mV/s for 2000 cycles

Figure 7 shows the cycling performance of the NiCo_2_O_4_@XMoO_4_ (X = Ni, Co) composite electrodes, which was evaluated through 2000 cycles with a scan rate of 60 mV/s. After 2000 cycles, it is found that the total capacitance retention was ~95.5 % for the NiCo_2_O_4_@CoMoO_4_ electrode and ~83.1 % for the NiCo_2_O_4_@NiMoO_4_ electrode, respectively. Compared with the NiCo_2_O_4_@CoMoO_4_ electrodes, the NiCo_2_O_4_@NiMoO_4_ electrode did not show a better cycling stability, but the characteristics of the high-rate capability and the large areal capacitance make the hierarchical heterostructures of the NiCo_2_O_4_@NiMoO_4_ a more prospective electrode material. The outstanding capacitive properties of the hierarchical heterostructures of the NiCo_2_O_4_@XMoO_4_ (X = Ni, Co) electrode are considered to originate from the synergistic effect of its following distinctive compositional and topological features [34–36]. First, within the hierarchical heterostructures, both core and shell are active materials, and the core-shell heterostructures enable easy access of electrolyte. Therefore, both of them can effectively contribute to the capacity. Secondly, the NiCo_2_O_4_ is highly conductive, which can provide “superhighways” for the charge in the core-shell structure. The direct growth of the XMoO_4_ nanosheets on the NiCo_2_O_4_ nanosheet arrays avoids the use of polymer binder/conductive additives and further guarantees the effective charge transport between them. Besides, the high electrical conductivity could decrease the charge transfer resistance of the electrodes, thus leading to an increased power density. Finally, the XMoO_4_ nanosheets and the NiCo_2_O_4_ nanosheets are mesoporous that increases the electroactive sites.

## Conclusions

In conclusion, 3D hierarchical heterostructures of the NiCo_2_O_4_@XMoO_4_ (X = Ni, Co) composite nanosheet arrays have been successfully designed and prepared for the supercapacitors. In such a novel nanostructure, the mesoporous NiCo_2_O_4_ nanosheet arrays grown directly on the Ni foam not only acted as a good pseudocapacitive material but also used as a hierarchical framework for loading NiMoO_4_ or CoMoO_4_ electroactive material. Notably, the NiCo_2_O_4_@NiMoO_4_ composite electrode showed excellent rate capability as well as a highest areal capacitance of 3.74 F/cm^2^ at 2 mA/cm^2^, which was much higher than the values for the NiCo_2_O_4_@CoMoO_4_ electrode (2.452 F/cm^2^) and NiCo_2_O_4_ electrode (0.456 F/cm^2^). The total capacitance retention of the NiCo_2_O_4_@CoMoO_4_ and NiCo_2_O_4_@NiMoO_4_ electrodes after 2000 cycles is ~95.5 and ~83.1 %, respectively. Based on these electrochemical properties, the NiCo_2_O_4_@NiMoO_4_ composite electrode material may be more appropriate for practical applications.
